# Maternal sevoflurane exposure induces temporary defects in interkinetic nuclear migration of radial glial progenitors in the fetal cerebral cortex through the Notch signalling pathway

**DOI:** 10.1111/cpr.13042

**Published:** 2021-05-06

**Authors:** Ming Jiang, Tianxiang Tang, Xinyue Liang, Juchen Li, Yue Qiu, Shiwen Liu, Shan Bian, Yunli Xie, Fang Fang, Jing Cang

**Affiliations:** ^1^ Department of Anesthesia Zhongshan Hospital Fudan University Shanghai China; ^2^ State Key Laboratory of Medical Neurobiology and MOE Frontiers Center for Brain Science Institutes of Brain Science Fudan University Shanghai China; ^3^ Institute for Regenerative Medicine School of Life Sciences and Technology Frontier Science Center for Stem Cell Research Shanghai East Hospital Tongji University Shanghai China

**Keywords:** interkinetic nuclear migration, neurogenesis, Notch, radial glial progenitor, sevoflurane

## Abstract

**Objectives:**

The effects of general anaesthetics on fetal brain development remain elusive. Radial glial progenitors (RGPs) generate the majority of neurons in developing brains. Here, we evaluated the acute alterations in RGPs after maternal sevoflurane exposure.

**Methods:**

Pregnant mice were exposed to 2.5% sevoflurane for 6 hours on gestational day 14.5. Interkinetic nuclear migration (INM) of RGPs in the ventricular zone (VZ) of the fetal brain was evaluated by thymidine analogues labelling. Cell fate of RGP progeny was determined by immunostaining using various neural markers. The Morris water maze (MWM) was used to assess the neurocognitive behaviours of the offspring. RNA sequencing (RNA‐Seq) was performed for the potential mechanism, and the potential mechanism validated by quantitative real‐time PCR (qPCR), Western blot and rescue experiments. Furthermore, INM was examined in human embryonic stem cell (hESC)‐derived 3D cerebral organoids.

**Results:**

Maternal sevoflurane exposure induced temporary abnormities in INM, and disturbed the cell cycle progression of RGPs in both rodents and cerebral organoids without cell fate alternation. RNA‐Seq analysis, qPCR and Western blot showed that the Notch signalling pathway was a potential downstream target. Reactivation of Notch by Jag1 and NICD overexpression rescued the defects in INM. Young adult offspring showed no obvious cognitive impairments in MWM.

**Conclusions:**

Maternal sevoflurane exposure during neurogenic period temporarily induced abnormal INM of RGPs by targeting the Notch signalling pathway without inducing long‐term effects on RGP progeny cell fate or offspring cognitive behaviours. More importantly, the defects of INM in hESC‐derived cerebral organoids provide a novel insight into the effects of general anaesthesia on human brain development.

## Graphical abstract

Maternal sevoflurane exposure during the neurogenic period temporarily induced abnormal INM of RGPs by targeting the Notch signalling pathway without inducing long‐term effects on RGP progeny cell fate or offspring cognitive behaviors. More importantly, the defects of INM in hESC‐derived cerebral organoids provide a novel insight into the effects of general anesthesia on human brain development.

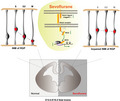

## 1 INTRODUCTION

Based on numerous studies in rodents and non‐human primates,^1^ the US Food and Drug Administration issued a warning that ‘repeated and lengthy use of general anaesthetics in children younger than three years or pregnant women during their third trimester may affect the development of children's brains’ (http://www.fda.gov/Drugs/DrugSafety/ucm532356.htm). Large‐scale clinical studies (GAS,^2^ PANDA,^3^ MASK,^4^ Canadian population‐based studies,^5^, ^6^ ALSPAC^7^ and the prospective clinical trial GAS)^8^ provided no evidence of clinically and statistically significant neurological defects in young children exposed to general anaesthetics. Controversy still exists regarding whether or how general anaesthetics affect neurodevelopment.^9^, ^10^ More importantly, these concerns might delay the necessary procedure and result in an adverse consequence.

It has been reported that 0.75%‐2% of pregnant women undergo non‐obstetric surgeries, and most of these surgeries are performed during the first two trimesters under general anaesthetics,^11^, ^12^, ^13^ which is a crucial period for neurogenesis.^14^ In mice, neurogenesis begins around embryonic day 10.5 (E10.5).^15^ Radial glial progenitors (RGPs), derived from neuroepithelial cells to form the ventricular zone (VZ) in the embryonic brain, give rise to most, if not all, pyramidal neurons. Unlike other progenitors, RGPs are bipolar cells with their apical processes anchored at the apical of the ventricle and basal processes to contact the basal lamina.^16^ The principal character of RGPs is interkinetic nuclear migration (INM), which describes the movement of nuclei along the apical‐basal axis synchronized with the cell cycle. In detail, nuclei move away from the apical surface during the G1 phase and stay at the basal side of the VZ during S phase. During the G2 phase, they return to the apical surface and undergo mitosis at the apical surface of the VZ.^17^ INM is vital for the efficient and continued production of neurons.^18^ Newborn pyramidal neurons migrate along the basal process of RGPs to form the six‐layered structure of the neocortex in an ‘inside‐out’ manner.^14^ The sequential generation of early‐born deep‐layer neurons followed by late‐born superficial‐layer neurons ensures the laminar organization of the mature neocortex during embryogenesis.^19^ Disruptions in neural progenitor maintenance and the balance between proliferation and differentiation have been shown to contribute to many neurodevelopmental disorders.^20^ Recently, INM was also proposed to underlie the pathogenesis of Huntington's disease and to be responsible for the abnormalities that occur in the developing cortex in Huntington's disease patients, including changes in mitosis and cell cycle progression.^21^

Clinically, sevoflurane is the most commonly used anaesthetic in pregnant women undergoing non‐obstetric surgery. Our previous studies on fetal brains have demonstrated that maternal sevoflurane exposure induces the abnormal proliferation of neural progenitors.^22^, ^23^ However, the potential mechanism remains elusive.

In this study, we found that maternal sevoflurane exposure transiently induced defects in the INM of RGPs during the peak of neurogenesis in the fetal brains via the Notch signalling pathway without eliciting long‐term effects. The same phenomena were observed in hESC‐derived cerebral organoids. These findings provide a novel insight into the effects of general anaesthetics on human brain development.

## 2 MATERIALS AND METHODS

### 2.1 Experimental animals

All procedures were approved by the Animal Care and Use Committee of Fudan University and followed institutional guidelines. Eight‐week‐old C57Bl/6 mice were obtained from the SLAC Laboratory. Animals were housed under controlled illumination (12 hours light/dark) and temperature (23‐24°C) with free access to food and water. Male and female mice were mated in a 1:2 ratio. The day of vaginal plug detection was defined as E0.5. Mouse embryos at E14.5 to E16.5 obtained from timely pregnant mice were used for experiments. For evaluating long‐term cognitive functions upon maternal sevoflurane exposure, two‐month‐old male mice were subjected to the Morris Water Maze (MWM) tests.

### 2.2 Human ESC culture and cerebral organoid culture

Human embryonic stem cells (hESCs) were obtained from WiCell, and cultured in a feeder‐free condition. Cells were maintained with mTeSR medium (Stemcell Technologies) on the Matrigel‐coated 6‐well plates at 37°C supplied with 5% CO_2_. Cells were cultured and passaged using standard procedures according to the previous description.^24^ Normal karyotype and contamination‐free were confirmed.

Cerebral organoids were cultured as a previous publication^25^ with slight modifications. Briefly, H9 hESCs were treated with 0.5 mmol/L EDTA and Accutase to obtain single‐cell suspension. Embryoid bodies (EBs) were generated with 9000 cells/well in the U‐bottom, Ultra low‐attachment 96‐well plates (Corning) with 150 µL of mTeSR medium containing 1xRevitaCell supplement (Gibco) at day 0. Fresh mTeSR medium without RevitaCell supplement was fed to EBs at day 3. At day 5, EBs were transferred into Neural Induction (NI) medium, and medium was exchanged with fresh NI medium every second day for 6 days. EBs were then embedded into Matrigel droplets, and cultured in differentiation medium without vitamin A and shaking. Five days later, cerebral organoids were cultured in differentiation medium supplied with vitamin A on an orbital shaker. Media were exchanged every 5 days until day 30, and used for further experiments.

### 2.3 Drugs and antibodies

Drugs and antibodies used in this study can be found in Table S1.

### 2.4 Animals anaesthesia

According to the previous protocol,^26^ minimum alveolar concentration (MAC) of C57Bl/6 mice was tested and 2.5% sevoflurane (approximately 0.9 MAC) was adopted in this study. At E14.5, E15.5 and E16.5, which is corresponding to the first two trimesters in human,^14^ the pregnant mice were randomly assigned into Control (Ctr) groups with 100% O_2_ exposure or sevoflurane‐treated groups (Sevo) with 2.5% sevoflurane exposure carried in 97.5% O_2_ for 6 hours (hrs) as previously.^23^ The mice in the Sevo group were anaesthetized in a box that was 20 × 30 × 20 cm^3^. A warm pad was used to avoid hypothermic. Arterial blood was sampled after 6 hrs of anaesthesia (data not shown) to guarantee the adequacy of ventilation and oxygenation. Caesarean sections were performed to extract embryonic brains at the end of O_2_/Sevoflurane treatment or 24 hrs after treatment.

### 2.5 Anaesthesia of hESC‐derived 3D cerebral organoids

Cerebral organoids were randomly assigned to Ctr group and Sevo group. Because the water/gas partition coefficient of sevoflurane is half lower than the blood/gas partition coefficient, the concentration of 4.1% sevoflurane was used in vitro instead of 2.5% in vivo as in our previous study.^22^, ^27^ In brief, the Sevo group was exposed to 4.1% sevoflurane in a 5% CO_2_ incubator, while the Ctr group was placed in another 5% CO_2_ incubator without anaesthesia. Both groups were incubated at 37°C for 6 hrs and then analysed after the treatment.

### 2.6 In utero electroporation (IUE)

IUE was performed according to a previous publication.^28^ Plasmids of pCAGEN‐SBP‐DICER1 (#50558), pCAGGS‐NICD (#26891) and pCAG‐GFP (#11150) were purchased from addgene. Mouse Jag1 was amplified and cloned into HindIII/BamHI sites of p3xFLAG‐CMT‐14 vector. Pregnant mice at E13.5 were anaesthetized with isoflurane (3% for induction and 2% during surgery for maintenance). A 1.5 cm incision was made along the linea alba in the lower abdomen, and the uterine horns were exposed. Desired plasmids (1.5 mg/mL) diluted in 1ul sterile Tris‐EDTA buffer (pH 7.4), which contained Fast Green (Sigma), were injected into the lateral ventricle of embryos at E13.5. Five 50 ms pulses of 33 V with 950 ms intervals were applied with a BTX electroporation system (ECM830). After electroporation, the uterine horns were placed back and the incision was sutured. The embryonic brains were used for further experiments at E15.5.

### 2.7 Immunofluorescence

Timely embryonic brains were fixed with 4% paraformaldehyde in PBS overnight and transferred to 30% sucrose in PBS for 24 hrs. Brains were embedded in tissue‐Tek OCT Compound (Sakura) and cryosectioned into 14 μm thickness. Cryosections were permeated in 0.5% Triton X‐100 in PBS for 30 minutes, and incubated with blocking solution (0.3% Triton X‐100, 5% normal donkey serum in PBS) for an hour at room temperature. After incubation with the primary antibody at 4°C overnight, sections were incubated with fluorescence‐conjugated secondary antibodies and DAPI (0.5 μg/mL in PBS, Sigma) for nuclei staining. Slices were mounted with aqua‐poly/mount (Polysciences). EdU staining was performed using the Click‐iT EdU Alexa Fluor^®^ 647 kit (Thermo Fisher Scientific) according to the manufacturer's instructions.

### 2.8 Western blot

Fetal cerebral cortex from both Ctr and Sevo groups at E14.5 was lysed in the lysis buffer and centrifuged at 13 000 rpm for 30 minutes at 4°C. The supernatant was collected and mixed with SDS‐PAGE Protein Loading Buffer (Yeasen), then boiled at 95°C and separated by 10% SDS‐polyacrylamide gel electrophoresis (PAGE). The proteins were transferred onto polyvinylidene difluoride membranes. The membranes were incubated with 5% non‐fat dry milk for 2 hrs at room temperature and primary antibody at 4°C overnight. After washed by TBST three times, the membranes were incubated with secondary antibody for 1 hr at room temperature, followed by washing with TBST three times. Blots were detected by ECL luminescence reagents (BBI) and imaged using ChemiDoc Imaging System (Bio‐rad). The bands were quantified by densitometry (ImageJ) to determine the expression of the protein. The ratio of band density of NICD over GAPDH was calculated.

### 2.9 Image acquisition and analysis

For statistics, at least three embryos from different pregnant mice were used in each group. Images were acquired by fluorescence microscopy (Nikon) and processed by NIS‐Elements AR (Nikon) and ImageJ. To analyse the distribution of BrdU+ ​or EdU+ ​cells, the VZ or the cortex was divided into 10 bins or 5 bins as described previously.^28^

### 2.10 RNA‐Seq and analysis

Total RNA was isolated from both Ctr group and Sevo group embryonic cerebral cortex at E14.5 using TRIzol reagent (Thermo Fisher Scientific) according to the manufacturer's instructions. RNA‐Seq was performed at Shanghai Majorbio Bio‐pharm Technology Co., Ltd. The data were analysed on the free online platform of Majorbio Cloud Platform (www.majorbio.com) and Metascape (metascape.org). Differential expression analysis was performed using DESeq2 with a cut‐off of FDR <.05 and abs (log_2_FC) >1.0. The heatmap and volcano plots were generated using R programming language, and the results of GO enrichment analysis were presented using Metascapse.

### 2.11 Quantitative real‐time PCR (qPCR)

Total RNA was isolated, and the cDNA was synthesized using Hifair III First‐Strand cDNA Synthesis Kit (Yeasen). QuantStudio 3 Real‐Time PCR Systems was used for quantitative real‐time PCR (Thermo Fisher Scientific) was used for qPCR with SYBR Green Master Mix (Yeasen). QuantStudio Design & Analysis Software (Thermo Fisher Scientific) was used for quantification with data normalized to the level of GAPDH mRNA. Each sample was measured in triplicate and the 2^−ΔΔ^ *^C^* ^t^ method was used. Primers used can be found in Table S2.

### 2.12 Morris water maze

Pregnant mice exposed to O_2_ or sevoflurane for 6hrs at E14.5 and the male adult offspring from both Ctr group and Sevo group (n = 20 in each group) were tested in MWM at P60 according to the published protocol.^29^ The young adult offspring were given training for five consecutive days (P60‐P64) and probed trails on the sixth day (P65). Mice were placed under a heated lamp for 5 minutes after each trial and then were put back to the regular cages. All tracks were recorded and analysed by EthoVision XT 8.5 (Noldus).

### 2.13 Statistical analysis

Data analysis and graphical presentation were performed using GraphPad Prism 8. Two‐tailed unpaired Student's *t* test was performed for comparison of two groups of data in this study. Two‐way ANOVA with repeated measurements with Bonferroni's *post hoc* was used to analyse the difference of escape latency in MWM and the distribution of BrdU‐positive or EdU‐positive cells in the VZ or in the cortex. At least three brain slices were analysed to obtain the mean number labelled by different neural markers per 100 μm surface length. Data are presented as mean ± SEM, and *P* < .05 was considered as statistically significant.

## 3 RESULTS

### 3.1 Maternal sevoflurane exposure impairs the INM of the mouse RGPs during the neurogenic period

In rodents, neurogenesis peaks at E14.5 and ceases at E16.5.^30^ To investigate the effects of sevoflurane on the RGPs during the neurogenic period, BrdU (50mg/kg) was administered into pregnant mice to label S phase RGPs at E14.5, E15.5 or E16.5 before they were treated with 6 hrs O_2_ or sevoflurane (Figure S1A). Surprisingly, BrdU+ ​RGPs in the Sevo groups were mainly located on the basal side of the VZ, while those in the Ctr groups were located on the apical side of the VZ (Figure [Link], [Link], [Link]B‐G). However, both Ctr and Sevo groups exhibited a similar number of BrdU labelled cells (Figure [Link], [Link], [Link]H).

**FIGURE 1:**
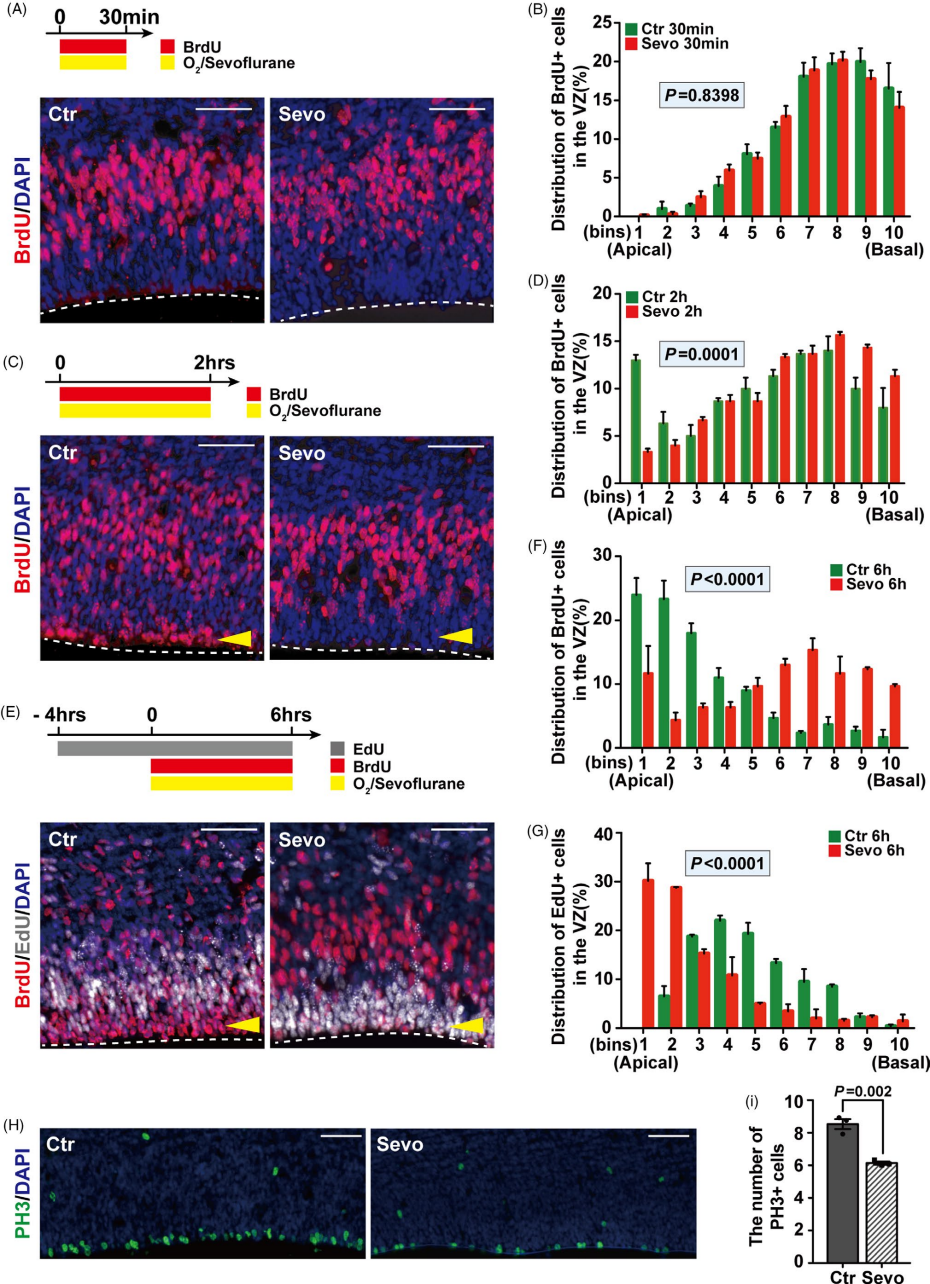
Maternal sevoflurane exposure impairs the INM of RGPs. (A, C, E) Representative images of sections from embryonic brains that were exposed to sevoflurane for 30 min (A), 2hrs (C) and 6hrs (E) stained for BrdU or EdU in both Ctr and Sevo groups. The VZ surface is outlined by a dashed line. (B, D, F, G) Quantification of the distribution of BrdU+ ​cells or EdU+ ​cells in each bin (the VZ was divided equally into 10 bins) after treatment for 30 min (B), 2hrs (D), 6hrs (F, G). Bin 1 starts from the apical surface. n = 3 for each group. (H) Representative images for PH3 in cortices of the Ctr and the Sevo groups. (I) Quantification shows the decreased number of PH3 + cells after sevoflurane exposure. n = 3 for each group. Data are presented as mean ± SEM. Scale bars represent 50 μm

The principal character of RGPs is INM, which plays an important role in neurogenesis.^31^ RGPs undergoing INM can be identified by thymidine analogues labelling: BrdU or EdU.^28^ To investigate the effects of sevoflurane on the basal‐apical INM progression, the localization of RGP nuclei was assessed 30 minutes, 2 hrs and 6 hrs after treatment with either O_2_ or Sevoflurane at E14.5 by the administration of BrdU to label cells in the S phase, the S‐G2 phase and the G2‐M phase, respectively (Figure 1A,C,E). We found that, after the exposure for 30 minutes which was equal to the S phase, most BrdU+ ​RGPs were located in the basal side of the VZ in both Ctr and Sevo cortices (Figure 1A,B). However, when some BrdU+ ​cells returned to the apical surface of the VZ in the Ctr group after treatment for 2 hrs, all the BrdU‐labelled RGPs in the Sevo group still stayed at the basal side of the VZ (Figure 1C,D). With the INM progression and duration of sevoflurane exposure extending to 6hrs, the majority of labelled cells returned to the apical side of the VZ in the Ctr group, while the most BrdU + cells were confined to the basal side of the VZ in the Sevo group (Figure 1E,F). To directly detect the progression of apical‐basal INM, the EdU (5 mg kg^‐1^) was injected 4 hrs before exposure to label cells in the M‐G1 phase (Figure 1E). Similarly, when the EdU‐labelled RGPs of the Ctr group moved basally, EdU+ ​cells in the Sevo group were arrested in the apical surface of the VZ (Figure 1G). In addition, the number of PH3 + cells (mitotic marker) decreased in the Sevo group (Figure H,I) which was further demonstrated that the nuclei failed to move. Taken together, our data indicate that the INM progression is impaired by maternal sevoflurane exposure.

### 3.2 Maternal sevoflurane exposure disturbs the cell cycle progression

**FIGURE 2:**
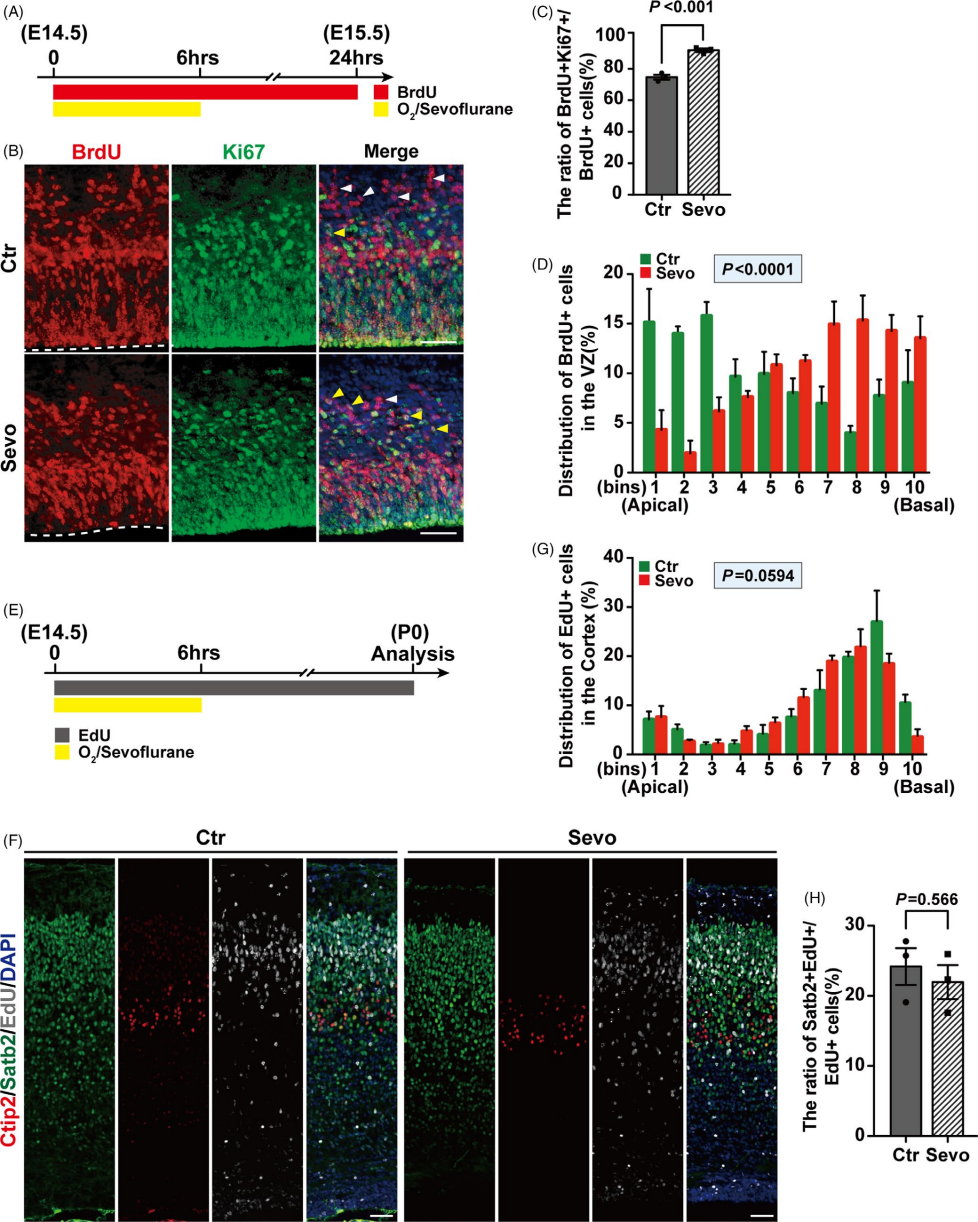
Maternal sevoflurane exposure disturbs cell cycle progression. (A, B) Representative images of sections from embryonic brains 24hrs after treatment on E14.5 (A) stained for BrdU and Ki67 (B). The VZ surface is outlined by a dashed line. (C) Quantifications show the ratio of BrdU+Ki67+ cells/BrdU+  cells was increased in the Sevo group compare to the Ctr group. The yellow arrows indicate the cells co‐labelled with BrdU and Ki67, while the white arrow only labelled with BrdU. n = 3 for each group. (D) Quantification of the distribution of BrdU+ ​cells in each bin (the VZ was divided as Figure 1). n = 3 for each group. (E) Schematic diagram of the timing of sevoflurane exposure at E14.5 and analysis at P0. (F) Representative images of embryonic cortices at P0 stained with EdU, Ctip2 and Satb2. (G) Quantification of the distribution of EdU+ ​cells in each bin (the cortex was divided equally into 10 bins) after prenatal treatment at P0. Bin 1 starts from the apical surface. n = 3 for each group. (H) Quantifications for the ratio of Satb2+EdU+  cells/EdU+ ​cells after prenatal treatment between the two groups at P0. n = 3 for each group. Data are presented as mean ± SEM. Scale bars represent 50 μm

INM is associated with the neurogenic process, which can influence RGP progeny.^31^ To determine the effects of impaired INM on neurogenesis, we used the proliferation marker Ki67 to label proliferating RGPs 24 hrs after treatment as shown in Figure 2A. Immunostaining of the embryonic cortex showed that the ratio of cells within the cell cycle (BrdU+Ki67+ cells /BrdU+ cells) increased (Figure 2B,C) in the Sevo group without a difference in the number of BrdU+ or Ki67+ ​cells (Figure S2A,B), which suggested that maternal sevoflurane induces cell cycle arrest. Besides, the distribution of BrdU+ ​cells in the VZ still maintained abnormal 24 hrs after treatment (Figure 2D).

RGPs undergo either symmetric divisions to expand the progenitor pool or asymmetric divisions to generate neurons and intermediate progenitors (IPs).^15^ The embryonic cortex was immunostained with the RGP marker Pax6 and the IP marker Tbr2 24 hrs after exposure (Figure [Link], [Link]). There was no significant difference in the number of RGPs or IPs between the two groups (Figure [Link], [Link]), suggesting that sevoflurane treatment did not alter the fate of progenitors during cortical development. To further confirm the cell fate of RGP progeny, RGPs in S phase were labelled with EdU (50 mg/kg) before treatment (Figure 2E). The cortices of offspring were immunostained with the deep‐layer neuron marker Ctip2^32^ and the superficial‐layer neuron marker Satb2^33^ at P0 (Figure 2F). We found the distribution of EdU+ ​cells in the whole cortex and the generation of superficial‐layer neurons showed no difference in Ctr and Sevo groups (Figure 2G,H and Figure S2H,I). Additionally, no Ctip2+EdU+ cells were found in neither group, which indicated that only superficial‐layer neurons were involved in maternal sevoflurane exposure at E14.5. These results suggest that although sevoflurane exposure during the neurogenic period leads to a disturbance in the cell cycle of RGPs, neurons generation is not affected.

### 3.3 Sevoflurane exposure impairs the INM of RGPs in the human cerebral organoids

**FIGURE 3:**
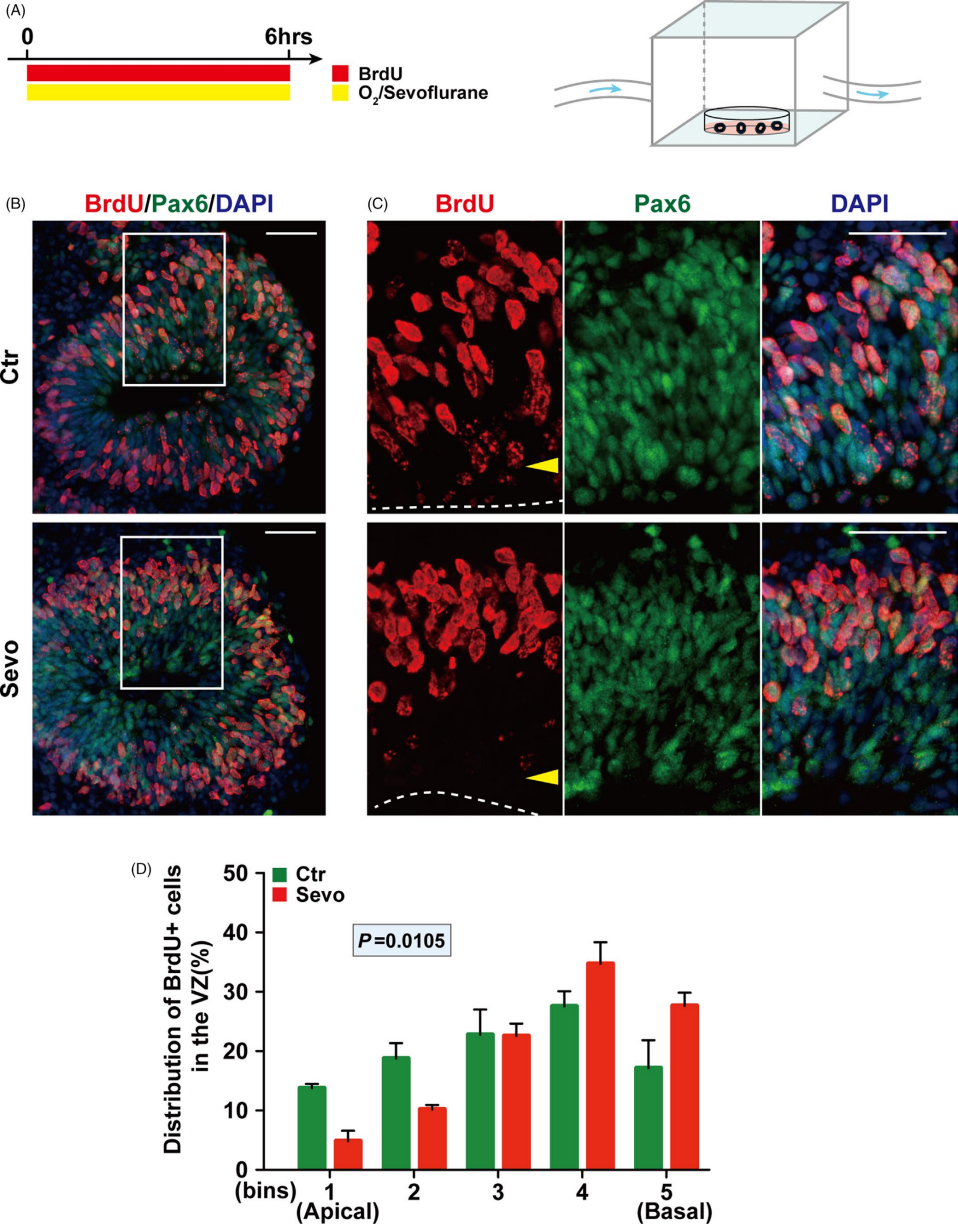
Sevoflurane exposure impairs the INM of RGPs in human cerebral organoids. (A) Schematic diagram of the timing of sevoflurane exposure and anaesthetic apparatus for cerebral organoid at day 30. (B) Representative images of sections from cerebral organoid after treatment stained with Pax6 and BrdU at day 30. (C) The surface of the VZ‐like structure is outlined by a dashed line. (D) Quantification of the distribution of BrdU+ ​cells in each bin. The VZ‐like structure determined by Pax6 + cells in the cerebral organoid section was divided equally into 5 bins. n = 3 for each group. Data are presented as mean ± SEM. Scale bars represent 50 μm

Organoids derived from pluripotent stem cells are able to self‐assemble to mimic early developmental processes.^34^ Previous studies showed that cerebral organoids display a similar organization to that of the developing human brain in the early stage.^25^ Hence, to understand the potential relevance of the rodent data described above to humans, hESC‐derived 3D cerebral organoids were used. On day 30, the cerebral organoids were pulsed with BrdU and treated with or without sevoflurane for 6hrs (Figure 3A). Pax6 was used as a marker of RGPs in the VZ‐like structure (Figure 3B,C). Importantly, we found a similar altered distribution of the BrdU‐labelling cells of Pax6+ ​RGPs (Figure 3D) as in rodents (Figure 1F) upon sevoflurane treatment. Therefore, our results indicate that sevoflurane exposure also impairs the INM of cerebral organoids derived from human embryonic stem cells.

### 3.4 Maternal sevoflurane exposure alters the expression of genes related to neurogenic progress

**FIGURE 4:**
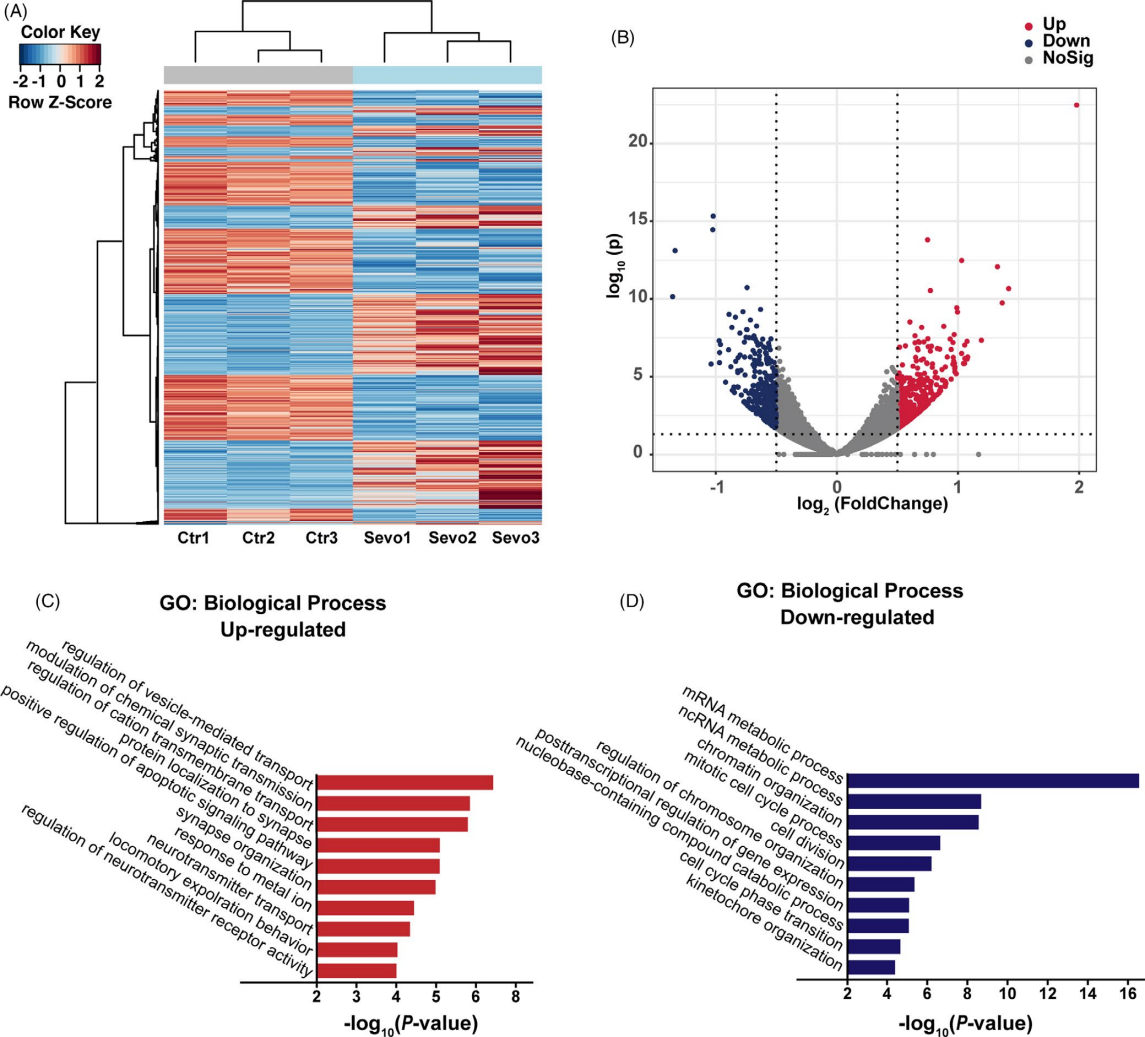
Maternal sevoflurane exposure alters the expression of genes related to neurogenic progress. (A) Heatmap of DEGs. (B) Volcano plots of DEGs. (C, D) GO enrichment analysis of the biological processes based on the RNA‐Seq data indicated the downregulated and upregulated DEGs. n = 3 for each group

RNA‐Seq analysis was performed to screen out the potential mechanism underlying the effect of maternal sevoflurane exposure on the INM of RGPs. Both heatmap and volcano plots (Figure 4A,B) showed that the gene expression profiles in the fetal cortex were indeed altered by sevoflurane. GO term analysis revealed that the upregulated genes (Figure 4C) were mainly enriched in signalling transport (for eg, regulation of vesicle‐mediated transport and regulation of cation transmembrane transport), while the downregulated genes (Figure 4D) showed the significant enrichment in biological processes were related to neurogenesis (for example, cell division, mitotic cell cycle process, cell cycle phase transition and kinetochore organization). Taken together, the GO enrichment analysis findings indicate that sevoflurane exposure indeed affected neurogenic progress.

### 3.5 The Notch signalling pathway is involved in the INM defects induced by sevoflurane

**FIGURE 5:**
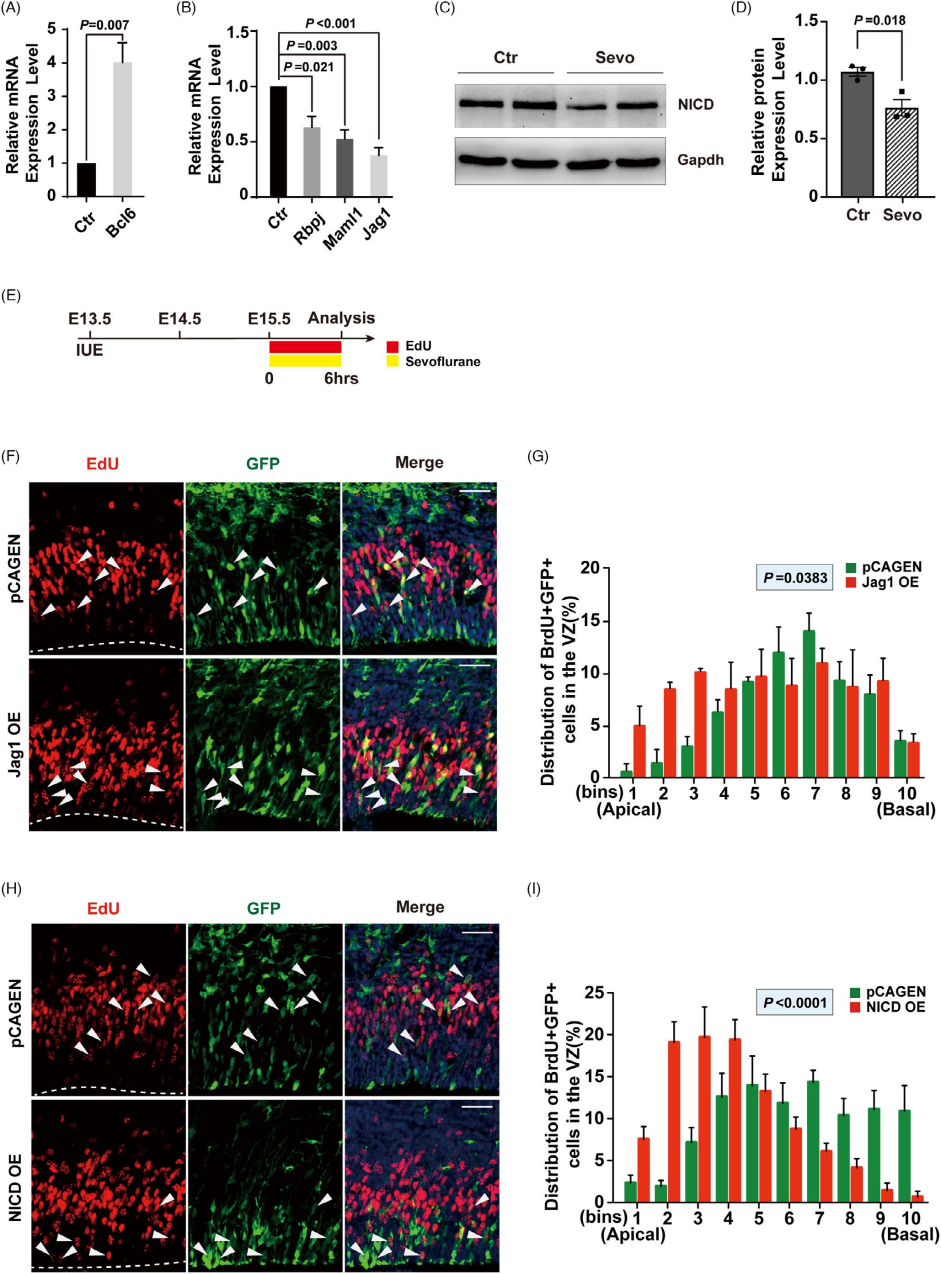
Notch signalling pathway is involved in INM defects induced by sevoflurane. (A, B) qPCR analysis to validate Notch‐related upregulated DEG. (A) and downregulated DEGs (B). n = 3 for each group. (C, D) The relative protein expression of NICD. n = 3 for each group. (E) Schematic diagram of the timing for IUE and sevoflurane exposure. (F, H) Representative images of sevoflurane exposed embryonic sections on E15.5 stained with GFP and EdU after IUE with NICD overexpression plasmid (F) or Jag1 overexpression plasmid (H) on E13.5. The VZ surface is outlined by a dashed line. The white arrows indicate EdU+ ​cells are co‐labelled with GFP. (G, I) Quantification of the distribution of EdU+ ​cells in each bin for (F, H) (the VZ was divided as Figure 1). n = 3 for each group. Data are presented as mean ± SEM. Scale bars represent 50 μm

Based on the RNA‐Seq analysis results, we performed qPCR on several differentially expressed genes (DEGs) that have been reported to be associated with embryonic neurodevelopment (Figure 5A,B and Figure [Link], [Link], [Link]). Among these genes, Bcl6 exhibited a nearly 4‐fold increase in expression after sevoflurane treatment, while the expression of Notch‐related genes—RBPJ, MAML1 and Jag1 was all downregulated by at least 30%. A recent study found that Bcl6 is a single cell‐intrinsic factor that ensures the robustness of neuronal fate transition^35^ by repressing multiple extrinsic pathways that promote self‐renewal, such as the Notch, Wnt and SHH signalling pathways. As a ligand of the Notch signalling pathway,^36^ Jag1 binds to the Notch receptor and triggers activation. The Notch intracellular domain (NICD) translocates to the nucleus, forms a complex with RBPJ binding protein and recruits co‐activators like MAML1 to promote the transcription of target genes.^36^ Moreover, we found the protein level of NICD decreased in the fetal cortex after sevoflurane exposure at E14.5 (Figure 5C,D). These results imply that the Notch signalling pathway is the potential mechanism underlying the impairment of INM induced by sevoflurane.

Therefore, to further confirm the role of the Notch signalling pathway in the effect of maternal sevoflurane exposure, plasmids expressing Jag1 and pCAGEN (as a control) were electroporated into RGPs *in utero*. Given the higher abortion rate of pregnant mice when IUE performed at E12.5 and the similar INM alternation when exposed to sevoflurane at E15.5 (Figure [Link], [Link], [Link]), we put off IUE to E13.5 and sevoflurane exposure to E15.5 EdU labelling was performed to track the INM of RGPs before exposure (Figure 5E). Our results showed the impairment of INM induced by sevoflurane was rescued in the Jag1‐overexpression group compared with the pCAGEN expression group (Figure 5F,G). Moreover, when NICD was overexpressed in the fetal cerebral cortex, the defect of INM was remarkedly attenuated (Figure 5H.I). Taken together, these data further provide compelling evidence that the Notch signalling pathway is involved in sevoflurane‐induced INM defects in the fetal cerebral cortex.

### 3.6 Maternal sevoflurane exposure does not affect the spatial learning or memory ability of young adult offspring

**FIGURE 6:**
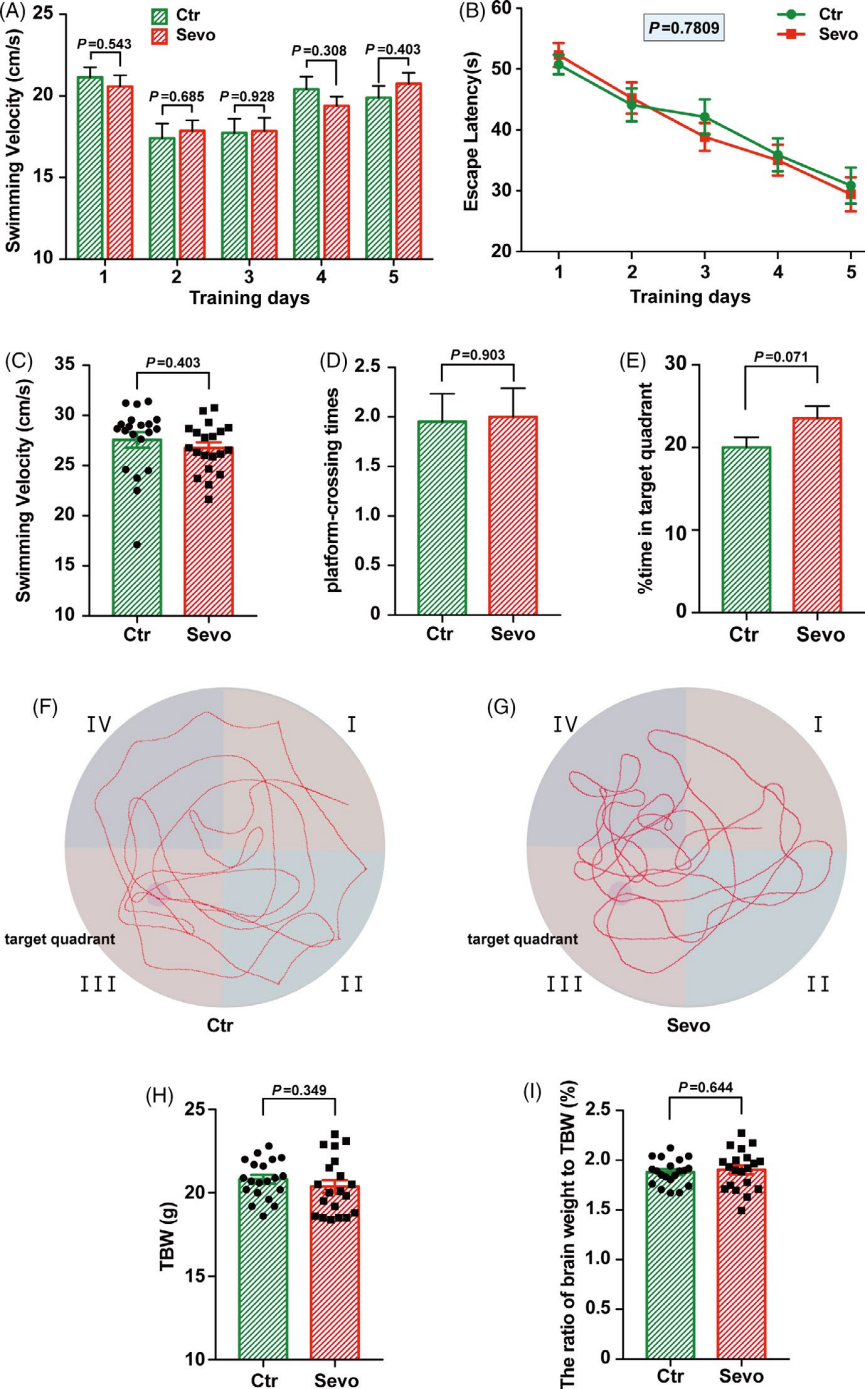
Maternal sevoflurane exposure did not affect spatial learning and memory ability in young male adult offspring. (A, B) Quantification of swimming velocity (A) and escape latency (B) in the Ctr and the Sevo groups during the training days. (C, D, E) Quantification of swimming velocity (C), platform‐crossing times (D) and percentage time in the target quadrant (E) in the two groups in the probe trails. (F, G) The representative trace of the Ctr and the Sevo groups when performed in the probe trails. (H, I) Quantification of TBW and the ratio of brain weight to TBW in the two groups. n = 20 for each group. Data are presented as mean ± SEM

We next planned to apply Morris water maze to examine the effect on the cognitive functions of young adult mice after maternal sevoflurane exposure. No statistically significant difference was observed between groups both in swimming speed (Figure 6A) and escape latency (Figure 6B) in trainning days. The platform‐crossing times and percentage of time in the target quadrant in the probe trials were also comparable between two groups (Figure 6C‐G). Additionally, the total body weight (TBW) and the ratio of brain weight to TBW (Figure 6H,I) had no difference between groups. These results indicate that the learning and memory abilities of young adult offspring remain intact after maternal sevoflurane exposure, suggesting that the alteration of INM upon maternal sevoflurane exposure has been recovered during brain development. However, the underlying mechanism needs to be uncovered in the future.

## 4 DISCUSSION

We provide a new perspective on the mechanism underlying the potential neurotoxicity of sevoflurane during the embryonic stage of brain development. Our results showed that during the peak of neurogenesis, the interkinetic nuclear migration of radial glial progenitors is transiently impaired both in the mice and in the hPSC‐derived 3D brain organoids after sevoflurane exposure. Furthermore, our data indicated that the Notch signalling pathway acts as a critical mechanism.

The most important finding in this study is the defect of INM in fetal brains after maternal sevoflurane exposure. INM is a character of pseudostratified epithelial cells such as the neural progenitor cells in the ventricular zone, which is an oscillatory nuclear movement in synchrony with the cell cycle.^17^ The aberration in apical‐basal‐INM and basal‐apical‐INM progression corresponded to an abnormal cell cycle progression (Figure 1A‐G) with prolonged cell cycle length and lower cell cycle exit rate (Figure 2C). Moreover, mitosis for securing self‐renewal of neural progenitors in the M phase^37^ was also decreased by sevoflurane exposure (Figure 1H,I). Interestingly, delayed mitosis has already been found in tissues outside the brain following anaesthesia exposure.^38^

Considering the difference between rodents and human beings, hESC‐derived 3D brain organoids were used in this study. Brain organoids have been widely used to explore the early development process^39^, ^40^ and are proved to be effective in the neurotoxicity‐related study in the developing brains.^41^ In this study, we observed a similar INM defects pattern in VZ‐like structure of 3D brain organoids after exposure to sevoflurane for 6hrs (Figure 3D) as that in the embryonic mouse brain (Figure 1F). This is a significant step for the study of general anaesthetics in human brain development.

In retinal neurogenesis, apical‐basal notch gradient is critical in neurogenesis regulated by interkinetic nuclear migration.^42^ When INM is perturbed, the nucleus is exposed to altered levels of Notch signalling, causing premature cell cycle exit and a temporal increase in neurogenesis which suggested the Notch acts as a key extrinsic pathway in nuclei movement. The results of RNA‐Seq analysis in this study and further qPCR and Western blot of Notch‐related DEGs did show an inhibition of Notch signalling in the fetal cortex after maternal sevoflurane exposure. Rescue experiments by overexpression of the Notch ligand—Jag1 or the activated Notch receptor—cleaved Notch1 (NICD) do attenuate the impairment of INM, especially in group NICD OE (Figure 5F‐I). Taken together, the Notch signalling is a potential mechanism underlying sevoflurane‐induced INM defects.

INM is believed to be a hallmark of vertebrate neural progenitors,^16^ which is vital for the efficient and continued generation of neurons.^18^ In our study, although the higher ratio for progenitors was temporarily arrested in the cell cycle and abnormal INM 24 hrs after treatment (Figure 2C,D), the proliferation and differentiation of neural progenitors (RGPs and IPs) were comparable after sevoflurane‐induced INM impairment (Figure 2E‐H, Figure [Link], [Link], [Link]), suggesting that INM is transiently affected by sevoflurane exposure without altering cell fate in progeny of RGPs.

Previous studies showed controversial behavioural outcomes following exposure to sevoflurane^23^, ^43^ with various concentrations, durations and experimental conditions. In this study, the learning and cognitive functions of young adult offspring were intact after maternal sevoflurane exposure with no difference in physiological development between the two groups. (Figure 6H,I). The results of MWM tests (Figure 6A‐G) were also consistent with the postnatal histological results. A recent study on the effects of maternal anaesthesia and surgery in rabbits showed a transient adverse effect on the offspring with delayed motor development in the first week of life and limited neurobehavioural impairment by 7 weeks age.^44^ Additionally, the Mayo Anesthesia Safety in Kids Study^4^ also showed no neuropsychological and behavioural defects in individuals aged 15‐20 years exposed to anaesthesia during childhood. Our study, along with above two researches, indicated an inspiring possibility that a self‐recovery mechanism exists. However, other studies in infant rhesus monkeys suggest that inhalation anaesthetics affect social behaviours, but do not impair specific cognitive domains,^45^, ^46^ which imply further investigations are needed to elucidate the potential neurotoxicity of inhalation anaesthetics on developing brains.

In conclusion, maternal sevoflurane exposure at the peak of neurogenesis transiently affects the INM of RGPs in the fetal VZ through the Notch signalling pathway but has no long‐term effect neurocognitive outcome. Maternal sevoflurane exposure is possibly safe for the neurodevelopment of its offspring.

## CONFLICT OF INTEREST

The authors declare that there are no conflicts of interest.

## AUTHOR CONTRIBUTIONS

JC, FF and YLX contributed to study design. MJ, TXT, XYL, JCL, SB and LSW contributed to experimentation. MJ and YQ contributed to data analysis. JC, FF, YLX and MJ contributed to final manuscript preparation.

## Supporting information

Fig S1

Fig S2

Fig S3

Table S1

Table S2

Supplementary Material

## Data Availability

The GEO accession number for the RNA sequencing data reported in this paper is GSE166607.

## References

[1] Lin EP , Lee JR , Lee CS , Deng M , Loepke AW . Do anesthetics harm the developing human brain? An integrative analysis of animal and human studies. Neurotoxicol Teratol. 2017;60:117‐128. 10.1016/j.ntt.2016.10.008 27793659

[2] Davidson AJ , Disma N , de Graaff JC , et al. Neurodevelopmental outcome at 2 years of age after general anaesthesia and awake‐regional anaesthesia in infancy (GAS): an international multicentre, randomised controlled trial. Lancet. 2016;387(10015):239‐250. 10.1016/s0140-6736(15)00608-x 26507180 PMC5023520

[3] Sun LS , Li G , Miller TL , et al. Association between a single general anesthesia exposure before age 36 months and neurocognitive outcomes in later childhood. JAMA. 2016;315(21):2312‐2320. 10.1001/jama.2016.6967 27272582 PMC5316422

[4] Warner DO , Zaccariello MJ , Katusic SK , et al. Neuropsychological and behavioral outcomes after exposure of young children to procedures requiring general anesthesia: the mayo anesthesia safety in kids (MASK) study. Anesthesiology. 2018;129(1):89‐105. 10.1097/ALN.0000000000002232 29672337 PMC6008202

[5] O'Leary JD , Janus M , Duku E , et al. Influence of surgical procedures and general anesthesia on child development before primary school entry among matched sibling pairs. Jama Pediatr. 2019;173(1):29‐36. 10.1001/jamapediatrics.2018.3662 30398535 PMC6583453

[6] Graham MR , Brownell M , Chateau DG , Dragan RD , Burchill C , Fransoo RR . Neurodevelopmental assessment in Kindergarten in children Eexposed to general anesthesia before the age of 4 Years. Anesthesiology. 2016;125(4):667–677. 10.1097/aln.0000000000001245 27655179

[7] Walkden GJ , Gill H , Davies NM , Peters AE , Wright I , Pickering AE . Early childhood general anesthesia and neurodevelopmental outcomes in the avon longitudinal study of parents and children birth cohort. Anesthesiology. 2020;133(5):1007‐1020. 10.1097/ALN.0000000000003522 32898216

[8] Shukla A , Chowdhary V . Neurodevelopmental outcome at 5 years of age after general anaesthesia or awake‐regional anaesthesia in infancy (GAS): an international, multicentre, randomised, controlled equivalence trial. Acta Paediatr. 2019;108(11):2115‐2116. 10.1111/apa.14943 31418482

[9] Jevtovic‐Todorovic V . Detrimental effects of general anaesthesia on young primates: are we closer to understanding the link? Br J Anaesth. 2021;126(3):575‐577. 10.1016/j.bja.2020.12.019 33509616

[10] Baxter MG , Fehr T . Developmental exposure to general anaesthesia: missed connections? Br J Anaesth. 2021;126(4):756‐758. 10.1016/j.bja.2021.01.013 33581851

[11] Mazze RI , Kallen B . Reproductive outcome after anesthesia and operation during pregnancy: a registry study of 5405 cases. Am J Obstet Gynecol. 1989;161(5):1178‐1185. 10.1016/0002-9378(89)90659-5 2589435

[12] Goodman S . Anesthesia for nonobstetric surgery in the pregnant patient. Semin Perinatol. 2002;26(2):136‐145. 10.1053/sper.2002.32203 12005471

[13] Devroe S , Bleeser T , de Van Velde M , et al. Anesthesia for non‐obstetric surgery during pregnancy in a tertiary referral center: a 16‐year retrospective, matched case‐control, cohort study. Int J Obstet Anesth. 2019;39:74‐81. 10.1016/j.ijoa.2019.01.006 30772120

[14] Cadwell CR , Bhaduri A , Mostajo‐Radji MA , Keefe MG , Nowakowski TJ . Development and arealization of the cerebral cortex. Neuron. 2019;103(6):980‐1004. 10.1016/j.neuron.2019.07.009 31557462 PMC9245854

[15] Kriegstein A , Alvarez‐Buylla A . The glial nature of embryonic and adult neural stem cells. Annu Rev Neurosci. 2009;32:149‐184. 10.1146/annurev.neuro.051508.135600 19555289 PMC3086722

[16] Gotz M , Huttner WB . The cell biology of neurogenesis. Nat Rev Mol Cell Biol. 2005;6(10):777‐788. 10.1038/nrm1739 16314867

[17] Taverna E , Huttner WB . Neural progenitor nuclei IN motion. Neuron. 2010;67(6):906‐914. 10.1016/j.neuron.2010.08.027 20869589

[18] Murciano A , Zamora J , Lopez‐Sanchez J , Frade JM . Interkinetic nuclear movement may provide spatial clues to the regulation of neurogenesis. Mol Cell Neurosci. 2002;21(2):285‐300. 10.1006/mcne.2002.1174 12401448

[19] Okano H , Temple S . Cell types to order: temporal specification of CNS stem cells. Curr Opin Neurobiol. 2009;19(2):112‐119. 10.1016/j.conb.2009.04.003 19427192

[20] Ernst C . Proliferation and differentiation deficits are a major convergence point for neurodevelopmental disorders. Trends Neurosci. 2016;39(5):290‐299. 10.1016/j.tins.2016.03.001 27032601

[21] Barnat M , Capizzi M , Aparicio E , et al. Huntington's disease alters human neurodevelopment. Science. 2020;369(6505):787‐793. 10.1126/science.aax3338 32675289 PMC7859879

[22] Liu S , Fang F , Song R , Gao X , Jiang M , Cang J . Sevoflurane affects neurogenesis through cell cycle arrest via inhibiting wnt/beta‐catenin signaling pathway in mouse neural stem cells. Life Sci. 2018;209:34‐42. 10.1016/j.lfs.2018.07.054 30071197

[23] Song R , Ling X , Peng M , Xue Z , Cang J , Fang F . Maternal sevoflurane exposure causes abnormal development of fetal prefrontal cortex and induces cognitive dysfunction in offspring. Stem Cells Int. 2017;2017:6158468. 10.1155/2017/6158468 29098009 PMC5643154

[24] Bian S , Repic M , Guo Z , et al. Genetically engineered cerebral organoids model brain tumor formation. Nat Methods. 2018;15(8):631‐639. 10.1038/s41592-018-0070-7 30038414 PMC6071863

[25] Lancaster MA , Renner M , Martin CA , et al. Cerebral organoids model human brain development and microcephaly. Nature. 2013;501(7467):373‐379. 10.1038/nature12517 23995685 PMC3817409

[26] Dahan A , Sarton E , Teppema L , et al. Anesthetic potency and influence of morphine and sevoflurane on respiration in mu‐opioid receptor knockout mice. Anesthesiology. 2001;94(5):824‐832. 10.1097/00000542-200105000-00021 11388534

[27] Esper T , Wehner M , Meinecke CD , Rueffert H . Blood/Gas partition coefficients for isoflurane, sevoflurane, and desflurane in a clinically relevant patient population. Anesth Analg. 2015;120(1):45‐50. 10.1213/ANE.0000000000000516 25393590

[28] Tang T , Zhang Y , Wang Y , et al. HDAC1 and HDAC2 Regulate intermediate progenitor positioning to safeguard neocortical development. Neuron. 2019;101(6):1117‐1133. 10.1016/j.neuron.2019.01.007 30709655

[29] Vorhees CV , Williams MT . Morris water maze: procedures for assessing spatial and related forms of learning and memory. Nat Protoc. 2006;1(2):848‐858. 10.1038/nprot.2006.116 17406317 PMC2895266

[30] Kanski R , van Strien ME , van Tijn P , Hol EM . A star is born: new insights into the mechanism of astrogenesis. Cell Mol Life Sci. 2014;71(3):433‐447. 10.1007/s00018-013-1435-9 23907612 PMC11113452

[31] Kosodo Y . Interkinetic nuclear migration: beyond a hallmark of neurogenesis. Cell Mol Life Sci. 2012;69(16):2727‐2738. 10.1007/s00018-012-0952-2 22415322 PMC11115108

[32] Molnar Z , Cheung AF . Towards the classification of subpopulations of layer V pyramidal projection neurons. Neurosci Res. 2006;55(2):105‐115. 10.1016/j.neures.2006.02.008 16542744

[33] Britanova O , de Juan RC , Cheung A , et al. Satb2 is a postmitotic determinant for upper‐layer neuron specification in the neocortex. Neuron. 2008;57(3):378‐392. 10.1016/j.neuron.2007.12.028 18255031

[34] Pollen AA , Bhaduri A , Andrews MG , et al. Establishing cerebral organoids as models of human‐specific brain evolution. Cell. 2019;176(4):743‐756. 10.1016/j.cell.2019.01.017 30735633 PMC6544371

[35] Bonnefont J , Tiberi L , van den Ameele J , et al. Cortical neurogenesis requires bcl6‐mediated transcriptional repression of multiple self‐renewal‐promoting extrinsic pathways. Neuron. 2019;103(6):1096‐1108 e4. 10.1016/j.neuron.2019.06.027 31353074 PMC6859502

[36] Kopan R , Ilagan MX . The canonical Notch signaling pathway: unfolding the activation mechanism. Cell. 2009;137(2):216‐233. 10.1016/j.cell.2009.03.045 19379690 PMC2827930

[37] Ayeni JO , Audibert A , Fichelson P , Srayko M , Gho M , Campbell SD . G2 phase arrest prevents bristle progenitor self‐renewal and synchronizes cell division with cell fate differentiation. Development. 2016;143(7):1160‐1169. 10.1242/dev.134270 26893341

[38] Sturrock JE , Nunn JF . Mitosis in mammalian‐cells during exposure to anesthetics. Anesthesiology. 1975;43(1):21‐33. 10.1097/00000542-197507000-00004 1147307

[39] Del Dosso A , Urenda JP , Nguyen T , Quadrato G . Upgrading the physiological relevance of human brain organoids. Neuron. 2020;107(6):1014‐1028. 10.1016/j.neuron.2020.08.029 32970996 PMC10042151

[40] Chhibber T , Bagchi S , Lahooti B , et al. CNS Organoids: an innovative tool for neurological disease modeling and drug neurotoxicity screening. Drug Discov Today. 2020;25(2):456‐465. 10.1016/j.drudis.2019.11.010 31783130 PMC7039749

[41] Lee CT , Chen J , Kindberg AA , et al. CYP3A5 Mediates effects of cocaine on human neocorticogenesis: studies using an in vitro 3D self‐organized hPSC model with a single cortex‐like unit. Neuropsychopharmacology. 2017;42(3):774‐784. 10.1038/npp.2016.156 27534267 PMC5240177

[42] Del Bene F , Wehman AM , Link BA , Baier H . Regulation of neurogenesis by interkinetic nuclear migration through an apical‐basal notch gradient. Cell. 2008;134(6):1055‐1065. 10.1016/j.cell.2008.07.017 18805097 PMC2628487

[43] Zheng H , Dong Y , Xu Z , et al. Sevoflurane anesthesia in pregnant mice induces neurotoxicity in fetal and offspring mice. Anesthesiology. 2013;118(3):516‐526. 10.1097/ALN.0b013e3182834d5d 23314109 PMC3580035

[44] der Van Veeken L , der Van Merwe J , Devroe S et al. Maternal surgery during pregnancy has a transient adverse effect on the developing fetal rabbit brain. Am J Obstet Gynecol. 2019;221(4):355.e1–355. 10.1016/j.ajog.2019.07.029 31336075

[45] Neudecker V , Perez‐Zoghbi JF , Coleman K , et al. Infant isoflurane exposure affects social behaviours, but does not impair specific cognitive domains in juvenile non‐human primates. Br J Anaesth. 2021;126(2):486‐499. 10.1016/j.bja.2020.10.015 33198945 PMC8040121

[46] Raper J , De Biasio JC , Murphy KL , Alvarado MC , Baxter MG . Persistent alteration in behavioural reactivity to a mild social stressor in rhesus monkeys repeatedly exposed to sevoflurane in infancy. Br J Anaesth. 2018;120(4):761‐767. 10.1016/j.bja.2018.01.014 29576116 PMC6200105

