# Supplementary figures and images for: Maternal sevoflurane exposure induces temporary defects in interkinetic nuclear migration of radial glial progenitors in the fetal cerebral cortex through the Notch signalling pathway
Source: Cell Prolif. 2021 May 6;54(6):e13042. doi: 10.1111/cpr.13042 (PMC8168415; doi:10.1111/cpr.13042)

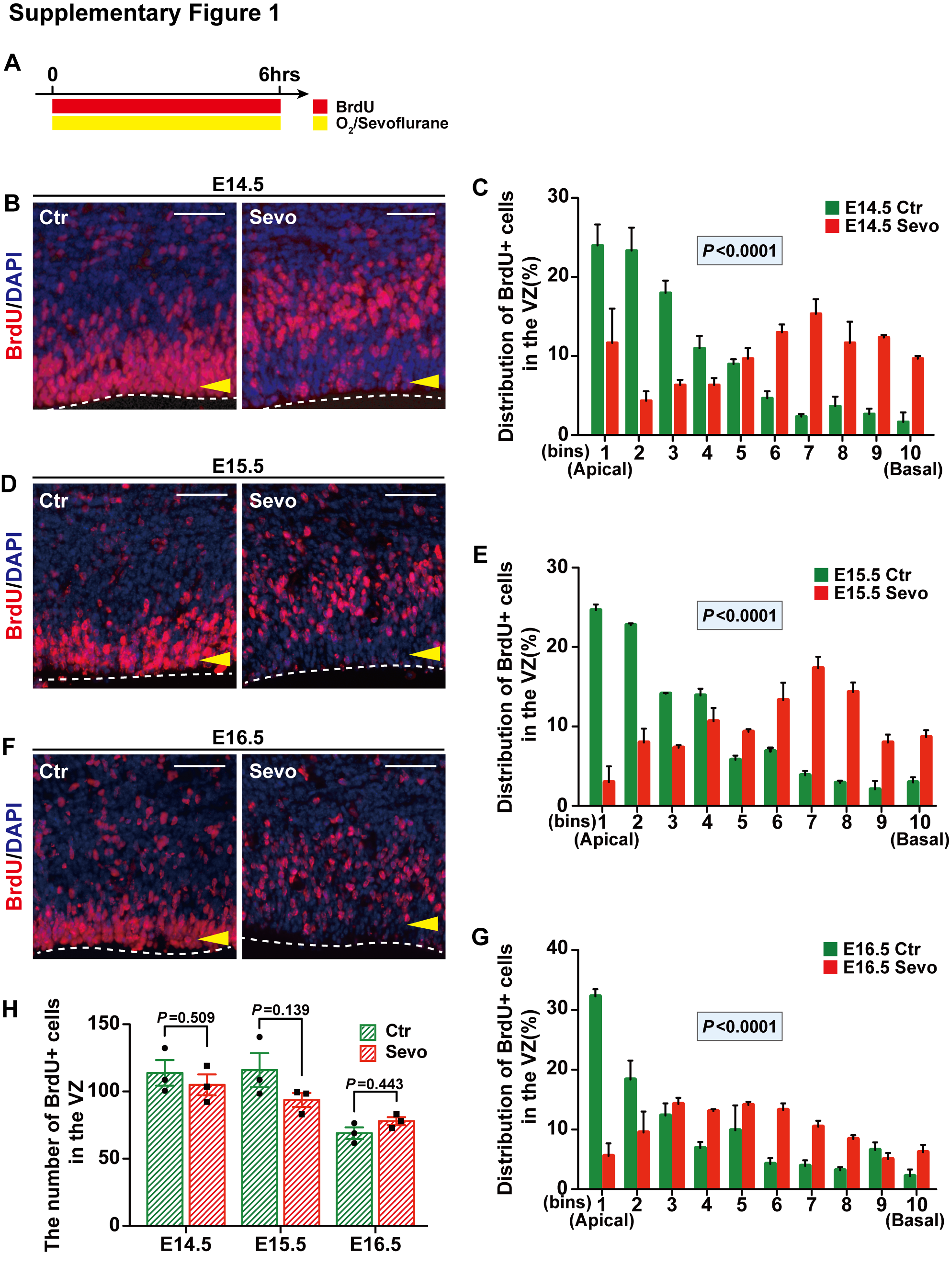

Supplement: Supplementary file 1 — Fig S1 [file CPR-54-e13042-s005.tif]

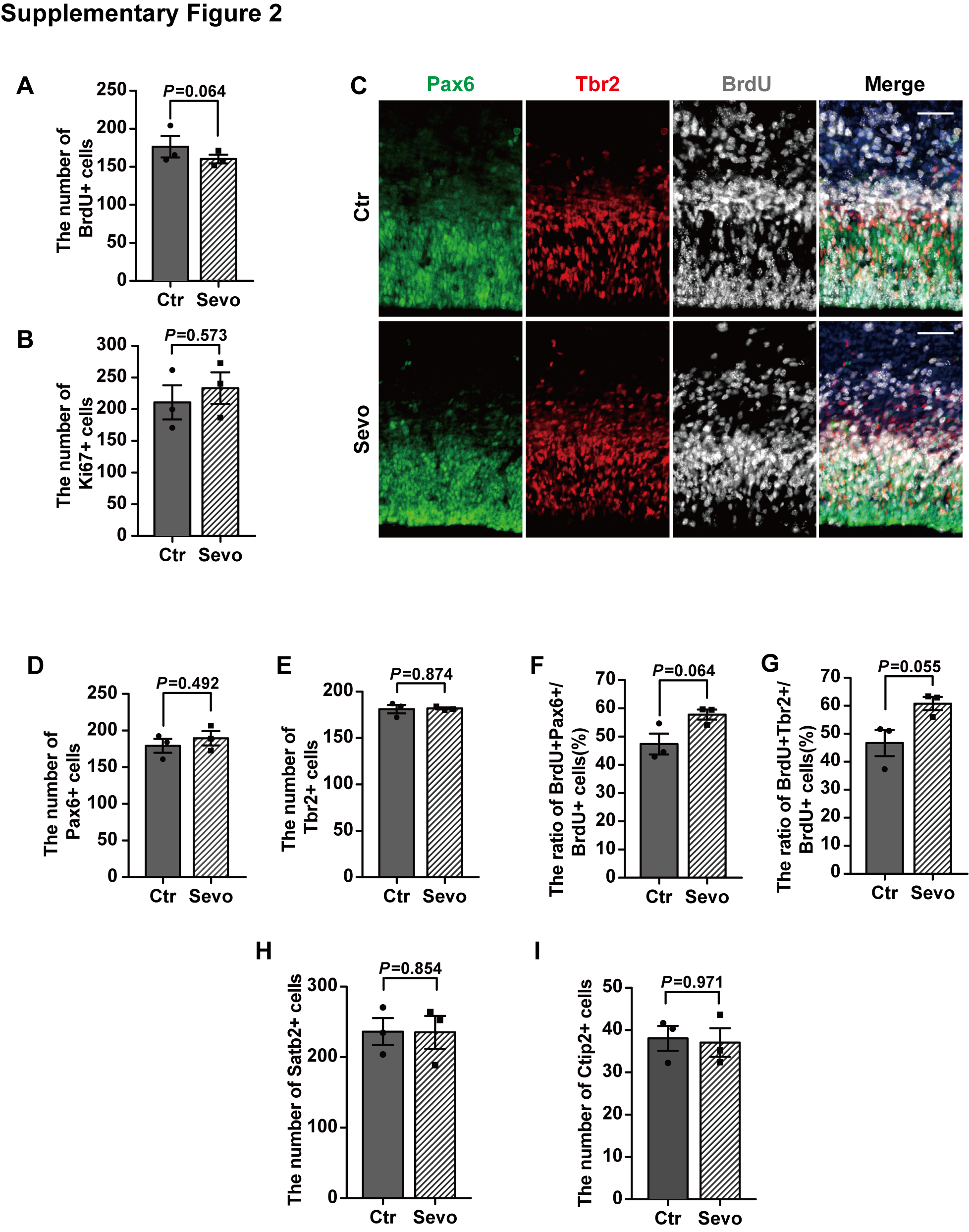

Supplement: Supplementary file 2 — Fig S2 [file CPR-54-e13042-s002.tif]

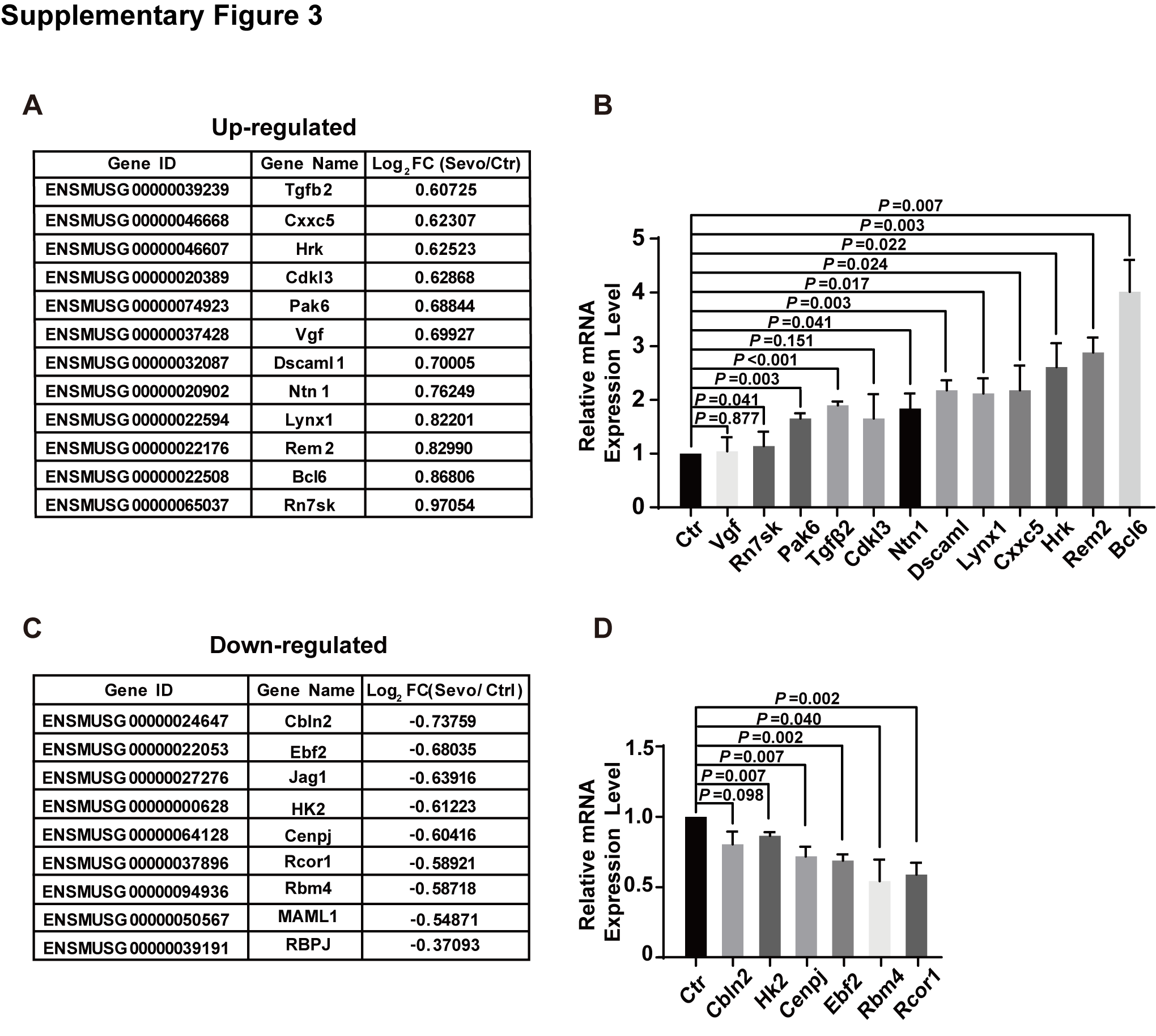

Supplement: Supplementary file 3 — Fig S3 [file CPR-54-e13042-s004.tif]

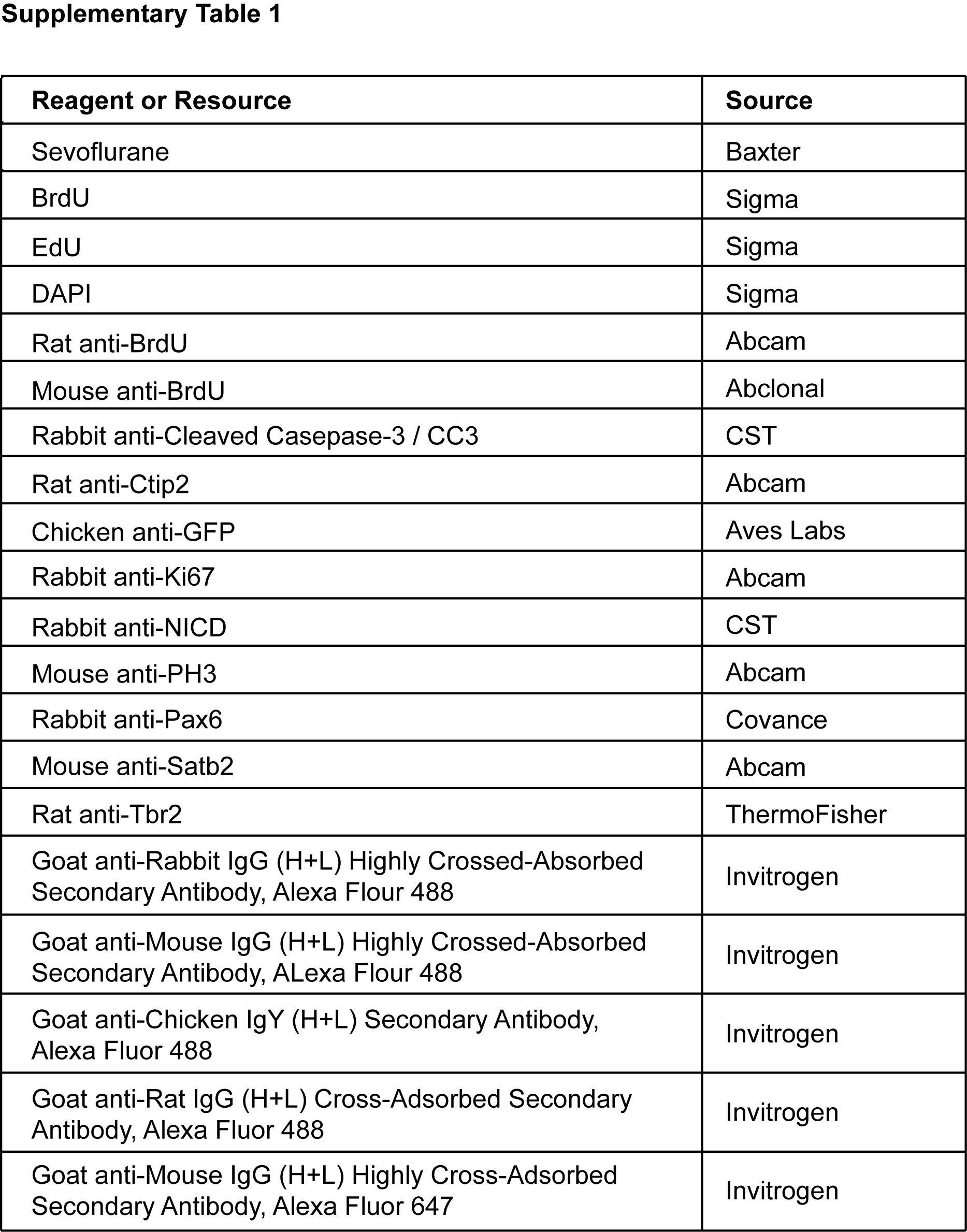

Supplement: Supplementary file 4 — Table S1 [file CPR-54-e13042-s003.tif]

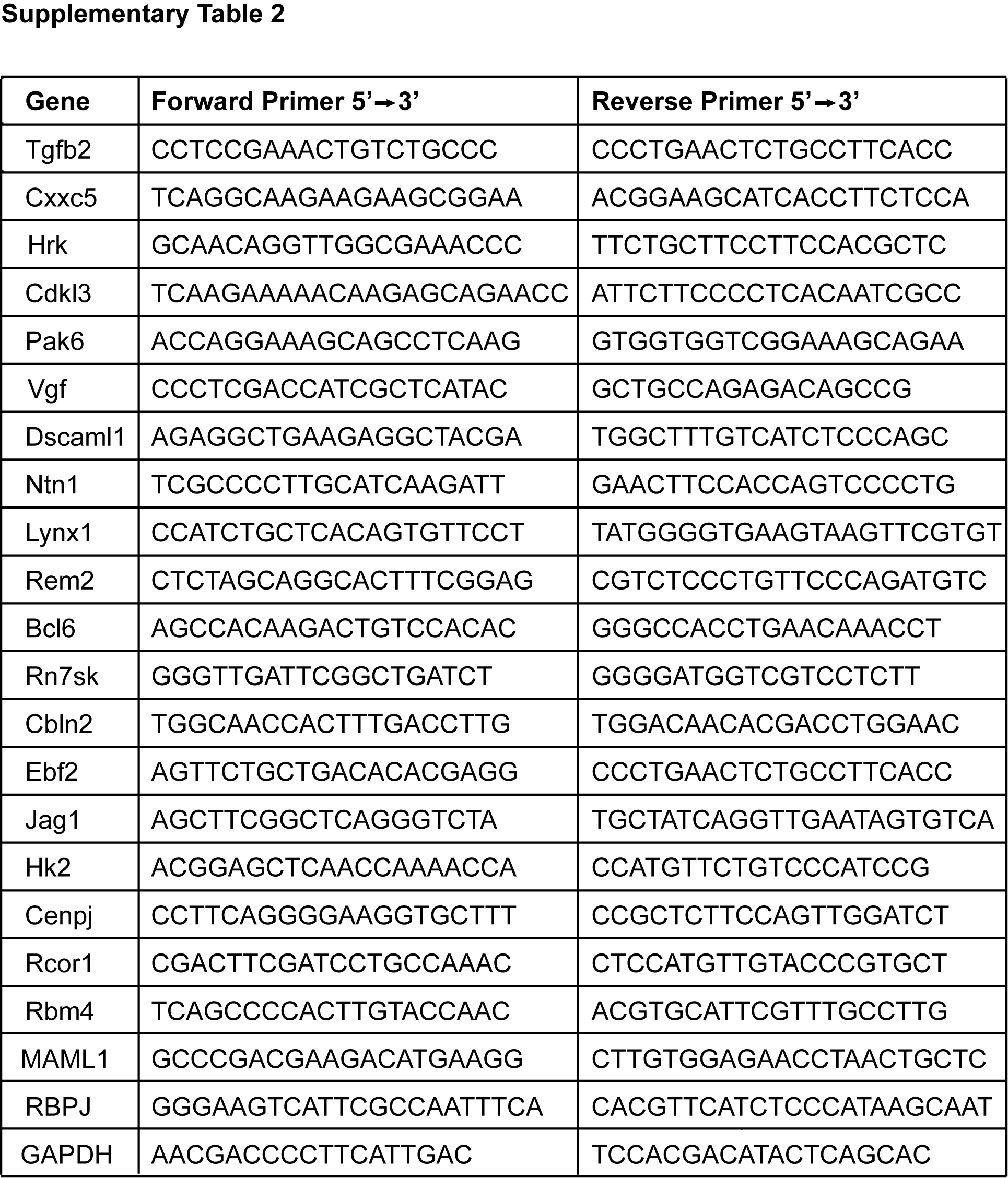

Supplement: Supplementary file 5 — Table S2 [file CPR-54-e13042-s001.tif]
