# Supplementary material for: Maternal sevoflurane exposure induces temporary defects in interkinetic nuclear migration of radial glial progenitors in the fetal cerebral cortex through the Notch signalling pathway
Source: Cell Prolif. 2021 May 6;54(6):e13042. doi: 10.1111/cpr.13042 (PMC8168415; doi:10.1111/cpr.13042)
Supplement: Supplementary file 6 — Supplementary Material [file CPR-54-e13042-s006.docx]

***Supplementary Information***

**Supplementary Table 1:** Reagents used in this study.

**Supplementary Table 2** Primers for RT-PCR used in this study.

***Supplementary Figure Legend***

**Supplementary Figure 1.** Maternal sevoflurane exposure induces alteration in RGP positioning during the neurogenic period. (A) Schematic diagram of the timing for sevoflurane exposure and analysis to assess the proliferation in the cortex. (B, D, F) Representative images of embryonic cortices from which were exposed to sevoflurane for 6hrs at E14.5 (B), E15.5 (D), and E16.5 (F) stained for BrdU in both Ctr and Sevo groups. The VZ surface is outlined by a dashed line. (C, E, G) Quantification of of BrdU+ cells distribution in each bin (the VZ was divided equally into 10 bins) at E14.5 (C), E15.5 (E) and E16.5 (G). Bin 1 starts from the apical surface. n=3 for each group. (H) Quantifications for the number of BrdU labeled cells on E14.5-E16.5. Data are presented as mean ± SEM. Scale bars represent 50μm.

**Supplementary Figure 2:** Maternal sevoflurane exposure did not affect upper-layer neurons generation and postnatal lamination in the cortex. (A, B) Quantifications for the number of BrdU+ cells and Ki67+ cells 24hrs after treatment in the Ctr and the Sevo group. n=3 for each group. (C) Representative images of embryonic cortices stained with BrdU, Pax6 and Tbr2 24hrs after treatment on E15.5. (D, E) Quantifications for the number of Pax6+ cells (D) and the number of Tbr2+ cells (E) 24hrs after treatment on E15.5 in the Ctr and the Sevo groups. n=3 for each group. (F, G) Quantifications for the ratio of Pax6+BrdU+/BrdU+ cells (F) and the ratio of Tbr2+BrdU+/BrdU+ cells (G) 24 hrs after treatment on E15.5 between the two groups. n=3 for each group. (H, I) Quantifications for the number of Satb2+ cells (H) and the number of Ctip2+ cells (I) after prenatal treatment in the Ctr and the Sevo groups at P0. n=3 for each group. Data are presented as mean ± SEM. Scale bars represent 50μm.

**Supplementary Figure 3:** Relative mRNA expression level of some neurogenesis-related DEGs after maternal sevoflurane exposure. (A, B) Some upregulated DEGs and downregulated DEGs that related with neurogenesis. (B, D) RT-PCR analysis to validate neurogenesis-related DEGs. n=3 for each group.
